# Malakoplakia of the colon following renal transplantation in a 73 year old woman: report of a case presenting as intestinal perforation

**DOI:** 10.1186/s13000-019-0799-z

**Published:** 2019-03-13

**Authors:** Andrew Mitchell, Alexandre Dugas

**Affiliations:** 1 Department of Anatomic Pathology and Cytology, Maisonneuve-Rosemont Hospital, 5415 Boulevard de L’Assomption, Montreal, QC H1T 2M4 Canada; 2 Department of Radiology, Maisonneuve-Rosemont Hospital, 5415 Boulevard de L’Assomption, Montreal, QC Canada

**Keywords:** Malakoplakia, Colon, Kidney, Renal, Transplant

## Abstract

**Background:**

Malakoplakia is a chronic inflammatory disease characterized by tissue infiltrates of large granular macrophages containing distinctive intracytoplasmic inclusions termed Michaelis-Gutmann (MG) bodies. The genitourinary system is the most commonly involved site, followed by the gastrointestinal tract. Malakoplakia may occur as a complication of primary or secondary immunosuppression and, therefore, renal transplant recipients are at risk. The graft itself or extra-renal sites may be involved. Regarding the latter, six cases of colorectal malakoplakia have been reported following renal transplantation, with all but one patient experiencing significant morbidity. We describe a further example of colorectal malakoplakia following renal transplantation. The other previously reported cases are reviewed.

**Case presentation:**

A 72 year old female presented with left lower quadrant abdominal pain and vaginal bleeding. She had received a cadaveric renal transplant for chronic renal failure ten months previously. Abdomino-pelvic computerized tomography (CT) scanning demonstrated two lesions in the mesocolon: the first adjacent to the descending colon and the second involving the sigmoid colon. A diagnosis of sub-acute perforated diverticulitis with two phlegmons was proposed. The sigmoid lesion was resected. The descending colon lesion was treated by creation of a cutaneous fistula. Microscopy of the sigmoid lesion showed the typical features of malakoplakia. She was discharged on sulfamethoxazole-trimethoprim. Nine months later, no longer receiving antibiotic therapy, the patient reported lower left quadrant discomfort. CT scanning showed para-rectal and pelvic abdominal masses with cutaneous and intestinal fistulas. Treatment with tazobactam-piperacillin was begun and sulfamethoxazole-trimethoprim was reinstated, with subsequent slow clinical improvement. Subsequent abdominal CT scans have shown persistence of the lesions.

**Conclusions:**

Physicians caring for renal transplant recipients should be aware of colorectal malakoplakia as a rare but serious complication. The onset may be within months or as long as a decade or more following transplantation. The clinical presentation is varied, nonspecific, and will likely suggest more common diseases. Although radiologic imaging is also nonspecific, awareness of malakoplakia is of importance to radiologists when formulating the differential diagnosis of mass lesions of the colorectum in this clinical setting. Definitive diagnosis remains dependent on pathologic examination of a biopsy or surgical resection specimen.

## Background

Malakoplakia is a rare chronic inflammatory disease characterized by tissue infiltrates of large granular macrophages-termed Hansemann histiocytes or von Hansemann cells-containing distinctive intracytoplasmic inclusions termed Michaelis-Gutmann (MG) bodies [1–4]. Hansemann histiocytes contain granular basophilic inclusions that vary from 5 to 15 mm and are periodic acid-Schiff positive, diastase resistant. Gram-negative bacteria may be visible. The histiocytes are positive with immunohistochemical stains against CD68, lysozyme and alpha-chymotrypsin [4]. Immunostaining with polyclonal anti-mycobacterium bovis has been described as a method of identifying organisms in malakoplakia [5]. MG bodies represent the calcified detritus of incompletely digested bacteria within phagolysosomes; although considered pathognomonic, they are not always present. They may be intracellular or extracelleular and are positive with the von Kossa stain for calcium and the Prussian blue stain for iron [4].

Theories regarding etiology include the role of microorganisms (of note, Eschericia coli is present in more than two thirds of cases), an abnormal immune response, and abnormal lysosome function within macrophages [1, 4].

The disease may involve any organ. The genitourinary system is the most commonly involved site (over 50% of cases), the bladder being most common, followed by the gastrointestinal tract [1, 2, 4]. There is a 4:1 female to male predominance for urinary tract involvement, but for other body sites there is no predisposition imparted by age, race and gender. Although the average age at presentation is 50 years, patients have ranged from 6 weeks to 85 years old [4]. Immunosuppresion (renal transplant, human immunodeficiency virus, tuberculosis, and cancer) is a factor in the development of a number of cases [3, 4].

Therapy of malakoplakia includes 1) antibiotic treatment using drugs able to concentrate within macrophages such as rifampicin, quinolone, and trimethoprim-sulfamethoxazole, 2) the cholinergic agonist bethanechol chloride to resolve the lysosomal defect, 3) tapering or withdrawal of immunosuppressive therapy, and 4) surgery [3, 4]. Prognosis is dependent on the location and extent of disease as well as the underlying health of the patient [3, 6–11].

As malakoplakia may occur as a complication of immunosuppression, whether primary or secondary, renal transplant recipients are at risk. The graft itself [12] or extra-renal sites may be involved [3]. Regarding the latter, six cases of colorectal involvement have been reported [6–11], with all but one patient experiencing significant morbidity. Herein, we describe a further example of colorectal malakoplakia following renal transplantation, in which the presenting features were at first suggestive of likely diverticular disease.

## Case presentation

### First hospital stay

A 72 year old female was seen for a regularly scheduled appointment at the renal transplant clinic of our hospital. Ten months previously she had received a cadaveric renal transplant (right lower flank) for chronic renal failure due to focal segmental glomerulosclerosis. Her immunosuppressive medications were prednisone, mycophenolate and tacrolimus. Six weeks prior to this visit she had been treated with valganciclovir for cytomegalovirus viremia. Now, she stated that she had recently begun to experience mild left lower quadrant abdominal pain and that vaginal bleeding had occurred the previous week. Fever was absent. Her bowel movements were unchanged and she did not report blood in her stool.

Her past medical history included iron deficiency anemia, arterial hypertension, diabetes type II, and hysterectomy with right ovariectomy for benign disease.

At physical examination the patient was afebrile with a mildly distended abdomen that was supple without tenderness or guarding. There was no palpable mass. The white blood cell count was 5.6 × 10^9^/L (reference: 4.5–10.8 × 10^9^/L). The hemoglobin was 94 g/L (reference: 123–157 g/L). Renal function was normal. She was admitted to hospital for further investigations.

**Fig. 1:**
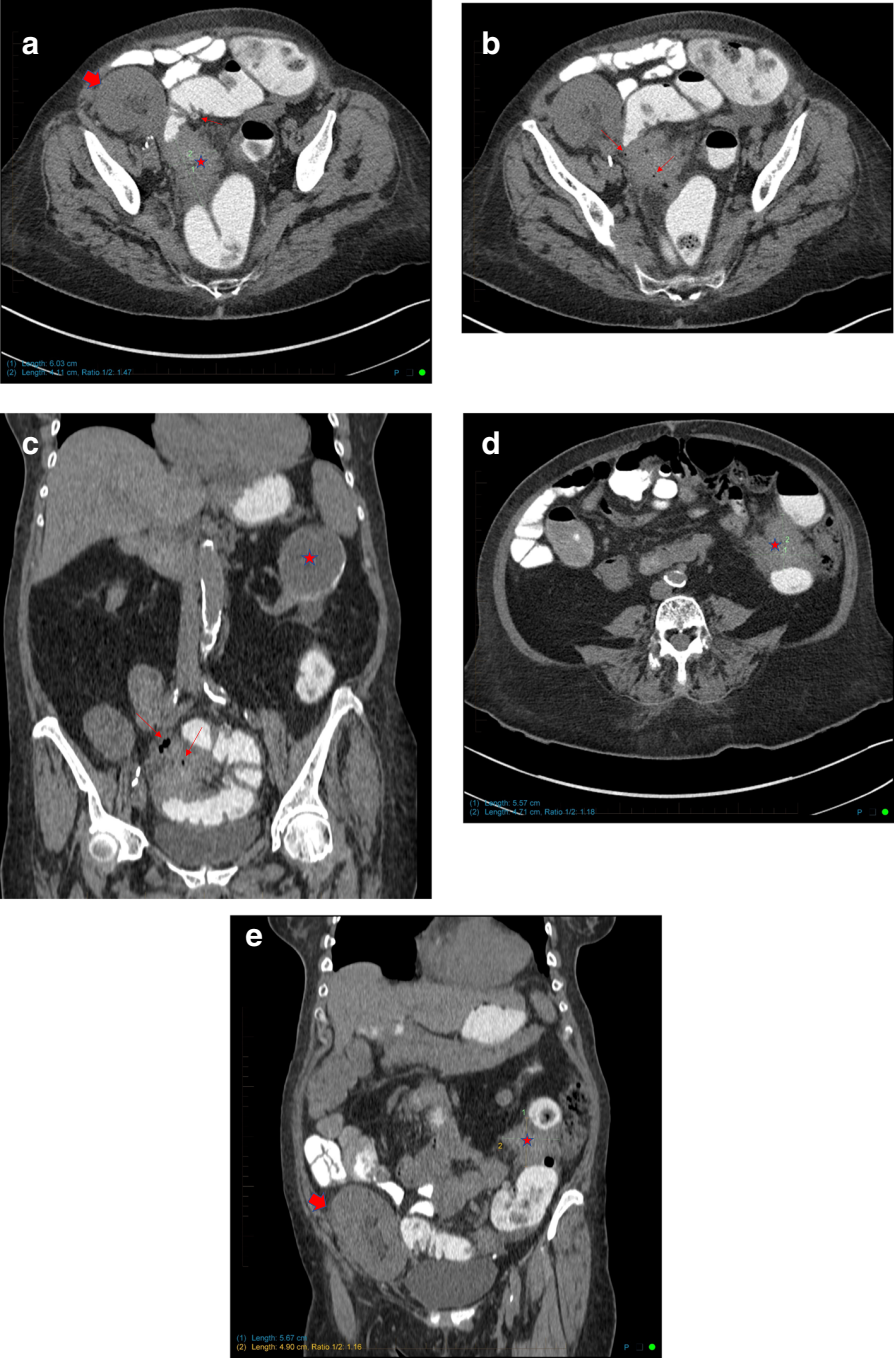
Abdomino-pelvic CT scan images. **a** Axial image showing a 6x4cm non stenotic mass (red star) adjacent to the distal sigmoid in a diverticular segment (thin red arrow). The transplanted kidney is partially seen in the right lower quadrant (thick red arrow). **b** and **c** Axial and coronal images showing small bubbles of extra-luminal air trapped in the mesosigmoid mass (red arrows). Incidentally, a partially calcified renal lesion is seen in the left native kidney (red star). **d** and **e** Axial and coronal images showing a second non stenotic mesocolic mass adjacent to the descending colon

Abdomino-pelvic computerized tomography (CT) scanning without intravenous iodine contrast was performed (Fig. 1). Rectal contrast was used to distend the colon. Two non-stenotic ill-defined moderately dense masses were found in the mesocolon: the first was adjacent to the descending colon, and the second involved the sigmoid colon As the second mass was in a diverticular bowel segment and contained few extraluminal air bubbles, a diagnosis of sub-acute perforated diverticulitis with two phlegmons was proposed. However, the findings were acknowledged as being somewhat atypical for this diagnosis because the proximal mass was completely separate from the distal one and contained no air. The differential diagnosis included a perforated sigmoid neoplasm with a metastatic implant next to the descending mesocolon and post-transplant lymphoproliferative disease. In light of these findings, a planned colonoscopy was canceled.

Given the presumptive diagnosis of perforated diverticular disease, empirical antibiotic therapy was begun and three days later laparotomy was performed. At surgery, two masses involving the descending colon and the sigmoid were found, the latter with perforation. The sigmoid lesion was resected and a terminal colostomy performed. The descending colon lesion was treated by creation of a cutaneous fistula and placement of a Jackson-Pratt drain.

**Fig. 2:**
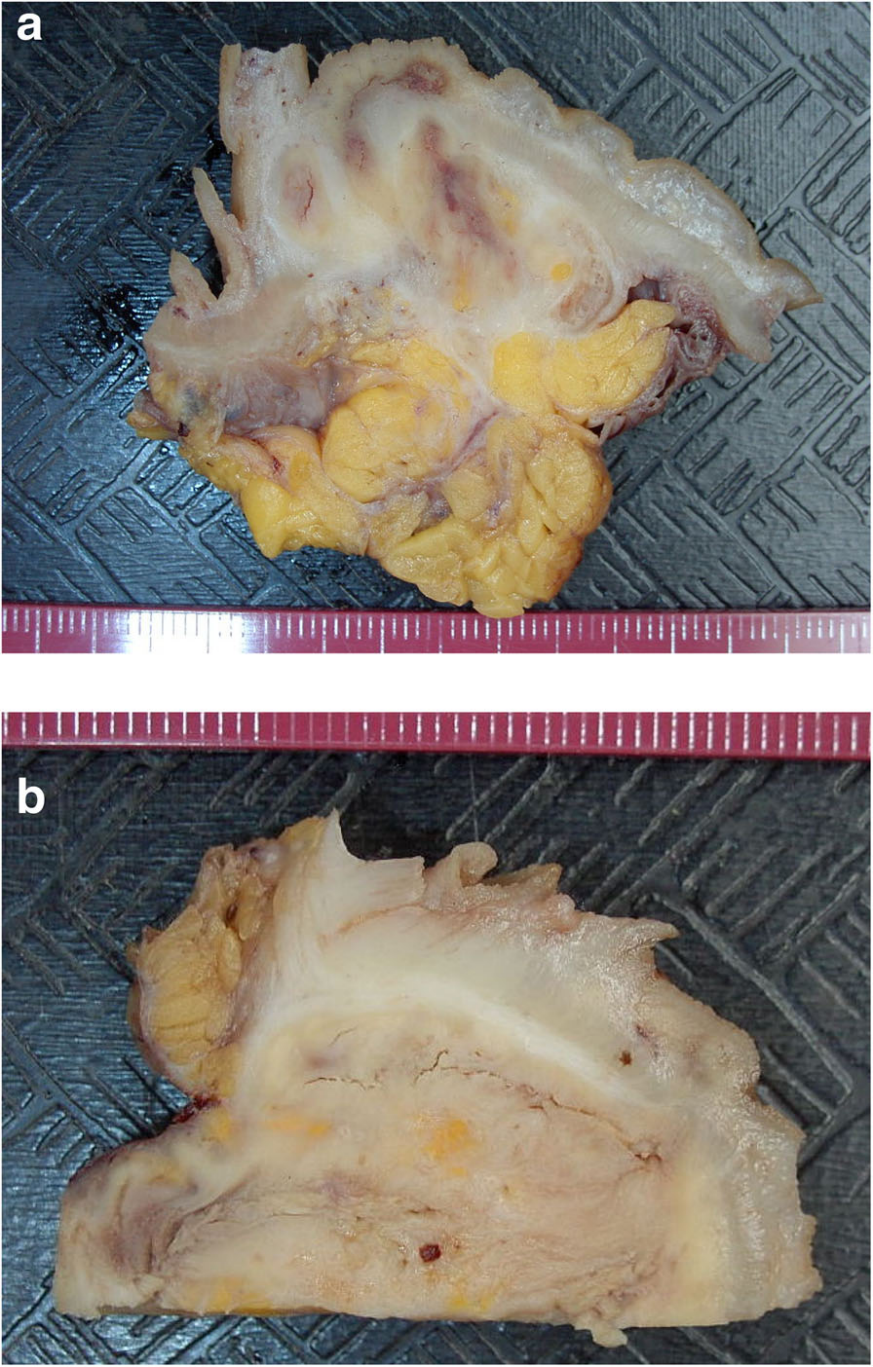
Macroscopy of the resected sigmoid. **a** and **b** In both images the mucosa is at top. The underlying bowel wall and mesentary are infiltrated and distorted by malakoplakia infiltrates which are friable and have visible cracking artefact

Macroscopic examination (Fig. 2) of the 26 cm long sigmoid specimen demonstrated a brownish serosa and a firm mesentery containing a friable, ill-defined mass 4,5 cm in diameter Multiple diverticula were also seen, without accompanying acute diverticulitis or abscess. There was no evidence of neoplasia.

**Fig. 3:**
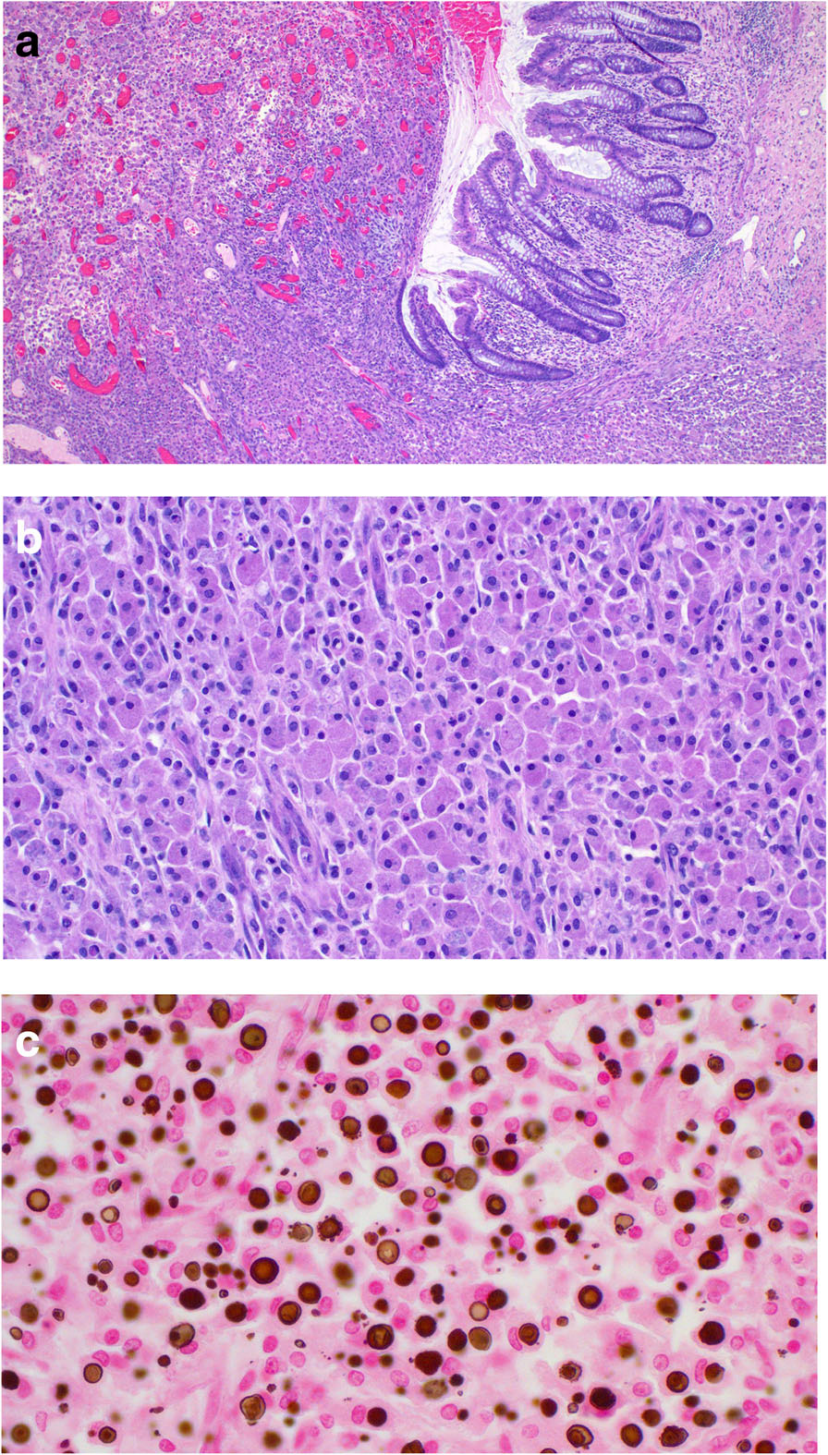
Microscopy of the resected sigmoid. **a** Low power image showing ulceration of the mucosa due to massive infiltration of all levels of the bowel wall by malakoplakia. **b** Medium power image of the sheet-like infiltrate of large non-atypical macrophages with abundant granular cytoplasm. **c** High power image of abundant MG bodies. They are of variable size with a round to ovoid shape. Many have a targetoid appearance

Microscopic examination (Fig. 3) showed a massive infiltrate of large non-atypical macrophages with abundant granular cytoplasm. The infiltrate involved the entire thickness of the bowel, causing mucosal ulceration and bowel wall perforation. Calculospherules (MG bodies) were readily identified in the cytoplasm. The cells were positive with the immunohistochemical markers leukocyte common antigen and CD68, confirming their histiocytic nature. The findings were diagnostic of malakoplakia.

The immediate postoperative course was uneventful. She was discharged home in good condition on sulfamethoxazole-trimethoprim 800/160 mg for twelve weeks.

### Second hospital stay

Nine months later, no longer receiving antibiotic therapy, the patient reported lower left quadrant discomfort and episodes of nausea. She had also noted a vulvar nodule which subsequent biopsy, as well as that of a vaginal mass discovered by pelvic examination, showed to be malakoplakia.

The following month she was admitted to hospital because of steadily worsening anal and pelvic pain, anorexia, weight loss, malnutrition, and an inability to walk unaided. She was afebrile. The colostomy site was unremarkable; the cutaneous fistula had a malodorous greenish discharge. There was no evidence of peritonitis. Renal function was normal. A CT scan showed para-rectal and pelvic abdominal masses with cutaneous and intestinal fistulas. Treatment with tazobactam-piperacillin was begun and sulfamethoxazole-trimethoprim 800/160 mg was reinstated, with subsequent slow clinical improvement. The patient was discharged home after several weeks.

Subsequent abdominal CT scans have shown persistence of the lesions. She is currently being closely followed without current antibiotic treatment.

## Discussion and conclusions

Malakoplakia involving the colorectum is a possible consequence of immunosuppression following renal transplantation. Although the small number of case reports suggests this is a rare complication, the one account of fortuitous diagnosis in an asymptomatic patient suggests the frequency is perhaps greater.

In general, the disease is characterized by significant morbidity. Patients may present in a variety of manners: acute abdomen due to bowel perforation (resulting in death in one instance), diarrhea, tenesmus, perianal pain, and painful defecation due to perianal fistula (Table 1). As the present case illustrates, bowel perforation may lead to a difficult and protracted clinical course.

**Table 1:** Reported Cases of Malakoplakia of the Colon Following Renal Transplantation

Case	Age/Sex	Time since transplantation	Underlying disease/Transplant Source	Site	Presentation	Treatment/Outcome
1. Kelleher (1990) [7]	62/M	18 months	CRF of unknown etiology/Cadaveric	Rectum and prostate	Tenesmus, perineal pain, dysuria	Antibiotics/Alive
2. Ourahma (1996) [8]	46/M	4 years	ADPKD/Cadaveric	Rectum	Diarrhea, Abdominal pain, fever	Antibiotics, reduced immuno-suppression, parasympathico-mimetics/Alive.
3. Berney (1999) [9]	52/M	9 years	ADPKD/Cadaveric	Ileo-cecum	Acute abdomen due to perforation	Right hemi-colectomy/Death from sepsis.
4. Yousif (2006) [10]	40/M	15 months	ADPKD/Live-related	Rectum, perianal region	Painful defecation, perianal fistula	Antibiotics, reduced immuno-suppression/Alive.
5. Shah (2010) [11]	45/M	3 years	Type 1 DM/Live-unrelated	Cecum, right colon, sigmoid	Watery diarrhea	Antibiotics, reduced immuno-suppression/Alive.
6. Bae (2013) [6]	55/F	11 years	IgA nephropathy/Live, relationship not specified	Ascending colon, transverse colon	Asymptom-atic	None/Alive.
7. Present Case (2018)	72/F	10 months	FSG/Cadaveric	Descending colon, sigmoid	Acute abdomen due to perforation	Antibiotics, sigmoidectomy/Alive.

*CRF* chronic renal failure, *ADPKD* Autosomal dominant polycystic kidney disease, *DM* Diabetes mellitus, *FSG* focal segmental glomerulosclerosis

Our case provides the second illustration of CT scan imaging of colorectal malakoplakia following renal transplant [7]. The images from these two cases suggest, however, that the CT scan findings of colorectal malakoplakia are nonspecific.

Treatment of malakoplakia is currently based on antibiotic therapy and reduction of immunosuppression. Surgery may be indicated for diagnosis and therapy when mass lesions are present. Although a discussion of specific treatment protocols is beyond the scope of this report, it is noteworthy that inadequate response to current protocols represents a major challenge in the treatment of certain patients.

We conclude that physicians caring for renal transplant recipients should be aware of colorectal malakoplakia as a rare but serious complication. The onset may be within months or as long as a decade or more following transplantation. The clinical presentation is varied, nonspecific and will likely suggest more common diseases. Although radiologic imaging is also nonspecific, awareness of malakoplakia is of importance to radiologists when formulating the differential diagnosis of mass lesions of the colorectum in this clinical setting. Definitive diagnosis remains dependent on pathologic examination of a biopsy or surgical resection specimen.

## Abbreviations

ADPKD: Autosomal dominant polycystic kidney disease
CRF: Chronic renal failure
CT: Computerized tomography
DM: Diabetes mellitus
FSG: Focal segmental glomerulosclerosis
MG: Michaelis-Gutmann
